# Prospective Trial of Neutrophil/Lymphocyte Ratio and Other Blood Counts as Biomarkers of Survival among Patients with High-Grade Soft Tissue Sarcomas Treated with Pegylated Liposomal Doxorubicin and Ifosfamide

**DOI:** 10.3390/cancers14143419

**Published:** 2022-07-14

**Authors:** Keith M. Skubitz, Evidio Domingo-Musibay, Bruce R. Lindgren, Edward Y. Cheng

**Affiliations:** 1 Department of Medicine, University of Minnesota Medical School, Minneapolis, MN 55455, USA; musib024@umn.edu; 2 Masonic Cancer Center, Minneapolis, MN 55455, USA; lindg001@umn.edu (B.R.L.); cheng002@umn.edu (E.Y.C.); 3 Department of Orthopaedic Surgery, University of Minnesota Medical School, Minneapolis, MN 55455, USA

**Keywords:** pegylated-liposomal doxorubicin, doxorubicin, sarcoma, lymphocyte, neutrophil, monocyte, platelet, ratio, dexamethasone, anti-emetic

## Abstract

**Simple Summary:**

Previous studies have reported an association between the ratio of neutrophils to lymphocytes circulating in blood and outcomes in patients with cancer. This study examined the association between lymphocyte and neutrophil counts and survival in a prospective trial of preoperative chemotherapy for high-grade soft-tissue sarcomas. A statistically significant association between overall survival was observed with the neutrophil/lymphocyte ratio. Our results suggest that a balance between the lymphocyte count and the number of circulating myeloid cells that might suppress lymphocyte function may be predictive of survival in patients with soft-tissue sarcomas. Future research should examine the role of lymphocyte-myeloid cell balance in sarcoma biology.

**Abstract:**

Several studies have reported an association between levels of circulating blood cells, in particular the neutrophil to lymphocyte ratio (absolute neutrophil count (ANC)/absolute lymphocyte count (ALC)) and outcomes in patients with cancer. In the current study, the association between lymphocyte, neutrophil, monocyte, and platelet counts and survival was examined in a prospective trial of preoperative pegylated-liposomal doxorubicin and ifosfamide for high-grade soft-tissue sarcomas. A statistically significant association between overall survival, but not progression free-survival, was observed with the ANC/ALC ratio at a cutoff value of ≥2 and a statistically significant trend using a cutoff of ≥5. Our results suggest that a balance between the lymphocyte count and the number of circulating myeloid cells that can suppress lymphocyte function may be predictive of survival in patients with soft-tissue sarcomas. Future research should therefore examine the role of lymphocyte-myeloid cell balance in sarcoma biology.

## 1. Introduction

The immune system has been recognized as having a potential role in sarcomas since the work of Coley in 1891 [1,2]. Inflammation plays an important role in many diseases including cancer, and many markers of inflammation can be quantified in the blood. A complete blood count is routinely performed in cancer, and several studies have suggested that measurements of different blood cells in the circulation can have predictive value in cancer.

Early lymphocyte recovery after the first course of chemotherapy, defined as absolute lymphocyte count (ALC) ≥ 500 at day 15, was reported to be a prognostic factor for overall survival in a retrospective study of 24 Ewing sarcomas [3]. Another retrospective study of 41 patients with Ewing sarcoma also found a positive association between day-15 ALC and survival [4]. Additional retrospective studies found that day-15 ALC ≥ 500 after the first cycle of chemotherapy predicted outcomes for other malignancies, including leukemia, breast cancer, and soft-tissue sarcoma (STS) [3,5,6]. A retrospective study of 19 patients with osteosarcoma treated between 1997 and 2007 found those with a higher day-14 ALC > 800 had longer overall survival [7]. A low pretreatment ALC was also correlated with poor outcomes in a variety of solid tumors ([8] and reviewed in [9]). Together, these studies suggest that lymphocyte count can be predictive of outcomes in a variety of sarcomas and other solid tumor malignancies.

Relative differences in populations of blood cells may also be important. The neutrophil/lymphocyte ratio (NLR), lymphocyte/monocyte ratio (LMR), platelet/lymphocyte ratio (PLR), and absolute monocyte count (AMC) have also been reported to be prognostic factors in malignancy [10]. A retrospective study of 83 patients with STS treated between 2002 and 2009 found a higher preoperative NLR in patients with STS compared with benign soft-tissue tumors; the higher preoperative NLR also had a negative effect on survival [11]. In one retrospective study of 55 osteosarcomas and 22 rhabdomyosarcomas treated between 2011 and 2015, investigators found that both an NLR ≥ 2 and ALC recovery were prognostic factors for survival of osteosarcoma and rhabdomyosarcoma [9]. Thus, several retrospective studies have found an association of NLR and survival in several types of sarcomas.

While NLR and ALC recovery have been found to be associated with survival in several malignancies, there are limited data on STS. In the present study, the relationships between ALC, ALC recovery, AMC, NLR, LMR, and PLR were examined for an association with overall survival in a prospective trial of preoperative pegylated liposomal doxorubicin (PLD) and ifosfamide in high-grade STS.

## 2. Methods

### 2.1. Study Design

This study examined 69 evaluable patients with STS receiving up to 4 cycles of preoperative chemotherapy with PLD and ifosfamide in a prospective clinical trial [12]. The relationships between the ALC, ALC recovery, AMC, NLR, LMR, and PLR were examined for an association with overall survival (OS) and progression-free survival (PFS). The study was approved by the University of Minnesota IRB. Patients were treated from 2006 to 2014 and were ≥16 years old with high-grade (FNCLCC grade 3) STS of the extremities or body wall whose tumors were greater than 5 cm in maximum diameter. Patients received preoperative chemotherapy followed by wide surgical excision of their tumor and subsequent external beam radiation; the goal of the clinical trial was to correlate treatment response with early PET changes. The current study represents a planned secondary endpoint. The chemotherapy regimen was PLD at 45 mg/m^2^ intravenously (IV) on day 1 every 28 days, with ifosfamide given by continuous intravenous infusion (CIVI) at 1.5 g/m^2^/day for 6 days (total dose over 6 days of 9 g/m^2^), in conjunction with mesna 1.5 g/m^2^/day for 7 days [12,13]. Granulocyte colony-stimulating factor (G-CSF) was used prophylactically.

### 2.2. Statistical Considerations

Demographics and patient characteristics were summarized by frequencies and percentages or medians and ranges (Table 1). The various individual blood counts or blood counts combined as ratios were evaluated in their original scale or dichotomized at certain cutoff values. Cox proportional hazard regression assessed the relationship between these blood count measures and the endpoints of PFS and OS. The estimated hazard ratio and 95% confidence intervals are reported in Table 2 and Table 3. There is one exception noted in Table 3 where the log-rank test was reported instead of Cox regression as a result of an over-inflated standard error due to only one event below the cutoff for NLR = 2. The time for PFS was calculated as the number of years from diagnosis to disease progression, relapse, or death from any cause. Patients alive without progression or relapse were censored at the date of last contact. OS was defined as the time from diagnosis to death from any cause with those still alive censored at the date of last follow-up.

This report addresses a secondary aim of the parent trial and, therefore, was not powered to find pre-determined effect sizes. The *p*-values presented in the tables have not been adjusted for multiple comparisons but are included to highlight possible important relationships between different blood cells, which can be used to generate hypotheses for a more definitive study. The 95% confidence intervals are also included in the tables to provide an additional measure for assessing the precision of these estimated effect sizes.

## 3. Results

Blood cell parameters were tested for an association with both PFS and OS. The total follow-up time ranged from 0.45 to 14.94 years, with a median of 8.06. A total of 37.7% of the patients were followed for more than 10 years. Of the 40 surviving patients alive at the last visit, 2 were lost to follow-up at 16 and 40 months. All other living patients were followed for a minimum of 5.3 years (median 11.2 years); 34 (89%) were followed for more than 8 years, 29 (76%) more than 9 years, and 23 (60%) for more than 10 years. Blood count was measured at pretreatment, before cycle 2 (analogous to cell recovery after one cycle), before surgery including all patients, and before surgery only including patients who received all four planned cycles of chemotherapy.

When the ALC was tested using a cutoff value of ≥800 or as a continuous variable, no association was observed between ALC at baseline, after one cycle of chemotherapy, or preoperatively for PFS or OS (Table 2 and Table 3). There were too few patients with cell counts below the cutoff values of ALC ≥ 500 and ALC ≥ 800 in the pretreatment group to perform statistical analysis.

When the ANC/ALC ratio was tested using cutoff values of 2 and 5, no association was observed for PFS (Table 2). However, a clear association with OS was observed for a cutoff value of ANC/ALC ≥ 2 (*p* = 0.039) and a trend using a cutoff of ≥5 (*p* = 0.069) (Table 3). No association was found in the other conditions tested, although there were too few patients with values below a cutoff for ANC/ALC ≥ 2 after cycle one and before surgery to perform statistical analysis.

When the AMC was tested as a continuous variable, an association with PFS was only seen after cycle 1 (*p* = 0.013). For OS, a significant association was observed after cycle 1 (*p* = 0.003) and a trend both at baseline (*p* = 0.105) and before surgery (*p* = 0.098) (Table 2 and Table 3). No statistically significant association was observed between the LMR or PLR and PFS or OS, although there was a trend toward association of LMR ≥2.4 at baseline with OS (*p* = 0.062) (Table 2 and Table 3).

## 4. Discussion

Several studies have suggested an association between the ALC, NLR, or other ratios between circulating leukocyte subsets and prognosis in sarcoma. In the present study, we found a statistically significant association between pretreatment NLR and OS using a cutoff value of ≥2 and a strong trend toward association with a cutoff value of ≥5 (*p* = 0.069). No association of the NLR at later times with OS was observed. No association of the NLR with PFS was observed. When analyzed as a continuous variable, there was a trend toward association with OS (*p* = 0.095). We used an NLR of 2 and 5 as cutoffs, although other studies have used various NLR cutoffs ranging from 2.3 to 5. This report addresses a secondary aim of the parent trial, and it was not powered to find pre-determined effect sizes. The p-values presented in the tables have not been adjusted for multiple comparisons but are included to highlight possible important relationships between different blood cells, which can be used to generate hypotheses for future studies. The 95% confidence intervals are included in the tables to provide a further measure of the precision of these estimated effects.

A retrospective study of 83 patients with STS treated between 2002 and 2009 found a higher preoperative NLR in patients with STS compared with benign soft tissue tumors and worse survival [11]. Nakamura also found an association between pretreatment NLR and disease-specific survival in a retrospective study of 142 patients with STS treated between 1995 and 2010 (49 were grade 1 and 23 grade 2; 129 were non-metastatic) [14]. A retrospective study of 260 STS cases, of which 43 were grade 1 using the Fédération Nationale des Centers de Lutte Contre le Cancer (FNCLCC) system, found an association of higher NLR and shorter time to both recurrence and OS (3.45 and 3.58 cutoffs, respectively) [15]. In another retrospective study, 340 STS cases (62 FNCLCC grade 1) were grouped into a training set (170) and a validation set (170) to determine cutoff values of NLR (<5); this study found an association between higher NLR and shorter OS [16]. Another retrospective study of 142 (9 grade 1, 13 grade 2) STS patients treated between 1964 and 2014 examined changes in NLR before and after initial treatment [17]. This study found that the NLR after treatment divided by the NLR before treatment (>1) was associated with OS.

Another retrospective study of 818 patients with localized STS (192 low grade) treated between 1994 and 2013 were split into a test group (403) and a validation group (415). This study found an association between NLR and elevated ANC, but not ALC, and survival [18]. Another retrospective study of 712 STS patients (56 grade 1, 67 grade 2) found that the NLR (defined cutoff >2.5) was associated with both worse OS and relapse-free survival in localized disease and OS in metastatic/unresectable disease [19]. Interestingly, this was not observed in FNCLCC grade 1 tumors [19]. Another retrospective study of 222 STS (65 grade 1) patients found the preoperative NLR (<2.5) was associated with OS [20].

Many earlier retrospective studies included lower-grade tumors as well as high-grade STS. It is possible that some associations of outcome with blood parameters could be at least partly dependent on tumor grade. For example, the study by Idowu et al. included a mixture of STS including lower-grade tumors, with only 40 FNCLCC grade-three sarcomas [11]. NLR was also associated with tumor grade and was higher in higher-grade tumors in one study [14].

Other retrospective studies have found an association between ALC recovery, AMC, LMR, and PLR and survival in STS [16,17,19,20]. In the current study, a trend toward an association of LMR at baseline with OS was observed (*p* = 0.060), but not at other times; no association with PFS was seen. We observed a slight trend toward an association of ALC before cycle 2 with both PFS and OS, although the potential effect was not large. The ALC before cycle 2 is a similar measure of lymphocyte recovery after cycle 1, which has been reported in other studies [3,4,5,6,7]. We also observed an association between AMC after cycle 2 with both PFS and OS. No association of PLR with PFS or OS was observed. Other markers of inflammation have also been associated with survival in STS such as C-reactive protein [14].

In contrast to most other studies, the strengths of our study are: (1) the prospective trial design yielding clinical data, (2) a uniform and well-defined preoperative chemotherapy regimen, (3) a homogeneous group of only high-grade STS excluding low- and intermediate-grade sarcomas, and (4) the long-term follow-up of all surviving patients. We found a statistically significant association of pretreatment NLR with OS in this group of patients with high-grade STS receiving preoperative chemotherapy with pegylated liposomal doxorubicin and ifosfamide. No clear association with PFS was observed, possibly due to the NLR serving as a marker of underlying tumor biology and behavior, rather than as a predictive marker of response to PLD and ifosfamide chemotherapy. Several hypotheses have been formulated regarding the mechanism whereby ALC and NLR may influence survival. For example, neutrophils have long been known to inhibit cytotoxic T cells and NK cells [21,22]. One mechanism of neutrophil-mediated lymphocyte suppression appears to be mediated by myeloperoxidase-generated free radicals [23]. Both neutrophils and monocytes can produce free radicals, Fas ligand, and TRAIL that could potentially suppress T-cell function, thus relating the NLR to the LMR [23,24,25]. Similarly, arginase-1 (ARG1)-expressing human granulocytic cells downregulate CD3ζ chains on T cells through L-arginine depletion, leading to inhibition of T-cell proliferation and cytokine secretion [26]. Currently, two types of myeloid-derived suppressor cells (MDSCs) are recognized that suppress immune responses in the tumor microenvironment. These cells consist of a subset of specifically activated neutrophils (PMN-MDSC) and monocyte-derived MDSCs (M-MDSC). These MDSCs can also stimulate other tumor activity, including growth, invasion, and angiogenesis. More recently, there is evidence that some cells that appear as monocytes in the circulation have the ability to transform into PMN-MDSCs [27]. A high NLR was associated with low LMR and high PLR in one study [19]. Our results suggest that a balance between the lymphocyte count and the number of circulating myeloid cells that can suppress lymphocyte function may be predictive of survival in patients with soft tissues sarcomas.

It is important to note that dexamethasone is both commonly used as an anti-emetic in cancer chemotherapy and is also highly lymphocytotoxic [28,29]. In addition, dexamethasone may protect some cancer cells from apoptosis [29]. Dexamethasone has a number of short- and long-term toxicities such as gastritis/ulcer, alteration of glucose metabolism, insomnia, psychiatric effects, avascular necrosis, muscle wasting, and bone loss. Some of these toxicities are not fully reversible, such as avascular necrosis and osteoporosis. The data on ALC in various cancers further suggest the widespread use of dexamethasone for nausea should be reconsidered unless truly necessary [30].

Our study confirms in a prospective trial with very long follow-up that the NLR correlates with survival in high-grade STS patients receiving preoperative chemotherapy. It is possible that this effect described in some retrospective studies could be more pronounced if lower-grade STSs were excluded. This effect could be mediated by neutrophil-derived free radical toxicity to lymphocytes, myeloid-derived suppressor cell effects, or another mechanism. Regardless of the mechanism, our results support a role for a balance between myeloid cells and lymphocytes in sarcoma and suggest an important role for this balance and lymphocyte function in sarcomas. Further, given the observed association of NLR and survival, it may be prudent to avoid unnecessary use of lymphocytotoxic agents such as steroids in STS.

## 5. Conclusions

This study found a statistically significant association of pretreatment NLR with OS in patients with high-grade STS receiving preoperative chemotherapy with pegylated liposomal doxorubicin and ifosfamide. The results support a role for a balance between myeloid cells and lymphocytes in sarcoma and suggest an important role for this balance and lymphocyte function in sarcoma biology. 

Dexamethasone is commonly used as an anti-emetic in cancer chemotherapy. In addition to the well-known short- and long-term toxicities, such as gastritis/ulcer, alteration of glucose metabolism, insomnia, psychiatric effects, avascular necrosis, muscle wasting, and bone loss, dexamethasone is also highly lymphocytotoxic. The data on ALC in sarcomas and other cancers further suggest the widespread use of dexamethasone for nausea should be reconsidered unless truly necessary.

## Figures and Tables

**Table 1:** Demographic Characteristics and Outcomes (N = 69).

Variable:	N	Mean (SD)	Median [Min/Max]
Age in years	69	52.5 (13.5)	54.0 [20/79]
**Variable**	**N**	**Percent (%)**	
Gender			
Male	42	62.9	
Female	27	39.1	
AJCC stage			
3	44	63.8	
4	25	36.2	
Disease status at last f/up			
Alive, no progression	34	49.3	
Alive, progression	13	18.8	
Dead of disease	22	31.9	

**Table 2:** Survival analysis of PFS by various cell count cutoff values at different times using Cox proportional hazard regression.

ALC *	Hazard Ratio (HR)	95% CI for HR	*p*-Value
**Model 1:** prior cycle 2 (n = 65) ALC ≥ 800	2.05	0.80, 5.28	0.136
**Model 2:** pre-surgery, all patients (n = 60) ALC ≥ 800	1.53	0.78, 3.01	0.220
**Model 3:** pre-surgery with all 4 cycles (n = 54) ALC ≥ 800	1.67	0.79, 3.54	0.181
**Model 4:** prior cycle 1 (n = 69) ALC (unit = 100 count increase)	1.01	0.94, 1.08	0.761
**Model 5:** prior cycle 2 (n = 65) ALC (unit = 100 count increase)	1.04	1.00, 1.07	0.053
**Model 6:** pre-surgery, all patients (n = 60) ALC (unit = 100 count increase)	1.05	0.97, 1.14	0.221
**Model 7:** pre-surgery with all 4 cycles (n = 54) ALC (unit = 100 count increase)	1.02	0.93, 1.12	0.679
ANC/ALC *	**Hazard Ratio (HR)**	**95% CI for HR**	***p*-Value**
**Model 1:** prior cycle 1 (n = 69) ANC/ALC ≥ 2	2.66	0.82, 8.61	0.104
**Model 2:** prior cycle 1 (n = 69) ANC/ALC ≥ 5	1.43	0.70, 2.93	0.327
**Model 3:** prior cycle 2 (n = 65) ANC/ALC ≥ 5	0.70	0.37, 1.35	0.287
**Model 4:** pre-surgery, all patients (n = 60) ANC/ALC ≥ 5	1.65	0.85, 3.22	0.138
**Model 5:** pre-surgery with all 4 cycles (n = 54) ANC/ALC ≥ 5	1.32	0.63, 2.76	0.468
**Model 6:** prior cycle 1 (n = 69) ANC/ALC ratio	1.03	0.93, 1.14	0.620
**Model 7:** prior cycle 2 (n = 65) ANC/ALC ratio	0.92	0.82, 1.03	0.135
**Model 8:** pre-surgery, all patients (n = 60) ANC/ALC ratio	1.04	0.96, 1.13	0.317
**Model 9:** pre-surgery with all 4 cycles (n = 54) ANC/ALC ratio	1.00	0.91, 1.10	>0.999
**AMC**	**Hazard Ratio (HR)**	**95% CI for HR**	***p*-Value**
**Model 1:** prior cycle 1 (n = 69) AMC (unit = 100 count increase)	1.05	0.95, 1.16	0.322
**Model 2:** prior cycle 2 (n = 64) AMC (unit = 100 count increase)	1.15	1.03, 1.28	0.013
**Model 3:** pre-surgery, all patients (n = 61) AMC (unit = 100 count increase)	1.01	0.90, 1.15	0.838
**Model 4:** pre-surgery with all 4 cycles (n = 54) AMC (unit = 100 count increase)	0.95	0.81, 1.10	0.463
LMR = ALC/AMC *	**Hazard Ratio (HR)**	**95% CI for HR**	***p*-Value**
**Model 1:** prior cycle 1 (n = 69) LMR ≥ 2.4	0.87	0.46, 1.64	0.661
**Model 2:** prior cycle 1 (n = 69) LMR continuous	0.94	0.70, 1.27	0.693
**Model 3:** prior cycle 2 (n = 64) LMR continuous	1.17	0.81, 1.69	0.393
**Model 4:** pre-surgery, all patients (n = 60) LMR continuous	1.25	0.77, 2.03	0.372
**Model 5:** pre-surgery with all 4 cycles (n = 54) LMR continuous	1.40	0.82, 2.40	0.220
**PLR = platelets/ALC**	**Hazard Ratio (HR)**	**95% CI for HR**	***p*-Value**
**Model 1:** prior cycle 1 (n = 69) PLR ≥ 135	1.67	0.65, 4.24	0.286
**Model 2:** prior cycle 1 (n = 69) PLR ≥ 200	0.96	0.51, 1.81	0.907
**Model 3:** prior cycle 2 (n = 64) PLR ≥ 200	1.04	0.41, 2.69	0.931
**Model 4:** pre-surgery, all patients (n = 60) PLR ≥ 200	0.94	0.41, 2.16	0.885
**Model 5:** pre-surgery with all 4 cycles (n = 54) PLR ≥ 200	0.86	0.35, 2.13	0.745
**Model 6:** prior cycle 1 (n = 69) PLR continuous (unit = 100 count increase)	0.94	0.69, 1.28	0.700
**Model 7:** prior cycle 2 (n = 64) PLR continuous (unit = 100 count increase)	0.85	0.69, 1.05	0.131
**Model 8:** pre-surgery, all patients (n = 60) PLR continuous (unit = 100 count increase)	0.96	0.80, 1.16	0.687
**Model 9:** pre-surgery with all 4 cycles (n = 54) PLR continuous (unit = 100 count increase)	0.88	0.70, 1.10	0.259

* There were too few patients with cell counts below the cutoff value for all ALC ≥ 500, and ALC ≥ 800 pre-treatment to perform a reliable statistical analysis. There are too few patients with cell counts below the cutoff for ANC/ALC ≥ 2 prior to cycle 2 and pre-surgery to perform a reliable statistical analysis. There were too few cases with values of LMR above the cutoff for LMR ≥ 2.4 prior to cycle 2 and pre-surgery, and too few cases with a PLR below the cutoff for PLR ≥ 135 prior to cycle 2 and pre-surgery to perform a reliable statistical analysis.

**Table 3:** Survival analysis of OS by various cell count values at different times using Cox regression.

ALC *	Hazard Ratio (HR)	95% CI for HR	*p*-Value
**Model 1:** prior cycle 2 (n = 65) ALC ≥ 800	1.49	0.51, 4.31	0.468
**Model 2:** pre-surgery, all patients (n = 60) ALC ≥ 800	1.81	0.80, 4.10	0.156
**Model 3:** pre-surgery with all 4 cycles (n = 54) ALC ≥ 800	1.67	0.70, 3.98	0.251
**Model 4:** prior cycle 1 (n = 69) ALC (unit = 100 count increase)	0.96	0.89, 1.04	0.342
**Model 5:** prior cycle 2 (n = 65) ALC (unit = 100 count increase)	1.04	1.00, 1.08	0.050
**Model 6:** pre-surgery, all patients (n = 60) ALC (unit = 100 count increase)	1.07	0.98, 1.17	0.129
**Model 7:** pre-surgery with all 4 cycles (n = 54) ALC (unit = 100 count increase)	1.02	0.91, 1.14	0.757
ANC/ALC *	**Hazard Ratio (HR)**	**95% CI for HR**	***p*-Value**
**Model 1:** prior cycle 1 (n = 69) ANC/ALC ≥ 2	6.27	na	0.039 †
**Model 2:** prior cycle 1 (n = 69) ANC/ALC ≥ 5	2.08	0.95, 4.57	0.069
**Model 3:** prior cycle 2 (n = 65) ANC/ALC ≥ 5	1.15	0.53, 2.50	0.733
**Model 4:** pre-surgery, all patients (n = 60) ANC/ALC ≥ 5	1.77	0.81, 3,88	0.155
**Model 5:** pre-surgery with all 4 cycles (n = 54) ANC/ALC ≥ 5	1.59	0.69, 3.69	0.278
**Model 6:** prior cycle 1 (n = 69) ANC/ALC ratio	1.09	0.99, 1.22	0.095
**Model 7:** prior cycle 2 (n = 65) ANC/ALC ratio	0.95	0.84, 1.08	0.412
**Model 8:** pre-surgery, all patients (n = 60) ANC/ALC ratio	1.02	0.94, 1.10	0.671
**Model 9:** pre-surgery with all 4 cycles (n = 54) ANC/ALC ratio	1.05	0.95, 1.16	0.387
**AMC**	**Hazard Ratio (HR)**	**95% CI for HR**	***p*-Value**
**Model 1:** prior cycle 1 (n = 69) AMC (unit = 100 count increase)	1.09	0.98, 1.20	0.105
**Model 2:** prior cycle 2 (n = 64) AMC (unit = 100 count increase)	1.21	1.07, 1.37	0.003
**Model 3:** pre-surgery, all patients (n = 61) AMC (unit = 100 count increase)	1.11	0.98, 1.26	0.098
**Model 4:** pre-surgery with all 4 cycles (n = 54) AMC (unit = 100 count increase)	1.03	0.88, 1.20	0.726
LMR = ALC/AMC *	**Hazard Ratio (HR)**	**95% CI for HR**	***p*-Value**
**Model 1:** prior cycle 1 (n = 69) LMR ≥ 2.4	0.50	0.24, 1.03	0.062
**Model 2:** prior cycle 1 (n = 69) LMR continuous	0.74	0.52, 1.06	0.105
**Model 3:** prior cycle 2 (n = 64) LMR continuous	1.12	0.70, 1.79	0.641
**Model 4:** pre-surgery, all patients (n = 60) LMR continuous	1.02	0.56, 1,83	0.960
**Model 5:** pre-surgery with all 4 cycles (n = 54) LMR continuous	1.08	0.57, 2.03	0.823
PLR = platelets/ALC *	**Hazard Ratio (HR)**	**95% CI for HR**	***p*-Value**
**Model 1:** prior cycle 1 (n = 69) PLR ≥ 135	1.36	0.48, 3.92	0.564
**Model 2:** prior cycle 1 (n = 69) PLR ≥ 200	1.07	0.51, 2.23	0.867
**Model 3:** prior cycle 2 (n = 64) PLR ≥ 200	0.91	0.31, 2.65	0.861
**Model 4:** pre-surgery, all patients (n = 60) PLR ≥ 200	0.91	0.34, 2.43	0.849
**Model 5:** pre-surgery with all 4 cycles (n = 54) PLR ≥ 200	0.73	0.27, 2.00	0.545
**Model 6:** prior cycle 1 (n = 69) PLR continuous (unit = 100 count increase)	1.02	0.72, 1.46	0.898
**Model 7:** prior cycle 2 (n = 64) PLR continuous (unit = 100 count increase)	0.84	0.65, 1.09	0.185
**Model 8:** pre-surgery, all patients (n = 60) PLR continuous (unit = 100 count increase)	0.87	0.70, 1.08	0.211
**Model 9:** pre-surgery with all 4 cycles (n = 54) PLR continuous (unit = 100 count increase)	0.89	0.69, 1.16	0.381

* There were too few patients with cell counts below the cutoff value for all ALC ≥ 500, and ALC ≥ 800 pre-treatment, and too few cases with cell counts below the cutoff for ANC/ALC ≥ 2 prior to cycle 2 and pre-surgery to perform a reliable statistical analysis. There are too few cases with LMR values above the cutoff for LMR ≥ 2.4 prior to cycle 2 and pre-surgery, and too few patients with PLR values below the cutoff for PLR ≥ 135 prior to cycle 2 and pre-surgery to perform a reliable statistical analysis. † The *p*-value is based on the log-rank test from the Kaplan-Meier method for estimating survival.

## Data Availability

Reasonable requests for other information will be honored.
